# The Discounted Money Value of Human Life Losses Associated With COVID-19 in Mauritius

**DOI:** 10.3389/fpubh.2020.604394

**Published:** 2020-11-10

**Authors:** Laurent Musango, Ajoy Nundoochan, Joses Muthuri Kirigia

**Affiliations:** ^1^World Health Organization, Country Office for Mauritius, Port Louis, Mauritius; ^2^African Sustainable Development Research Consortium (ASDRC), Nairobi, Kenya

**Keywords:** coronavirus, COVID-19, gross domestic product (GDP), human capital approach (HCA), value of human life

## Abstract

**Background:** Mauritius along with other 12 countries in the African Region was identified at the early start of the COVID-19 pandemic as being at high risk due to high volume of international travel, high prevalence of non-communicable diseases and co-morbidities, high population density and significant share of population over 60 years (16%). The objective of this study was to estimate the total discounted money value of human life losses (*TDMVCL*_*MAURITIUS*_) associated with COVID-19 in Mauritius.

**Methods:** The human capital approach (HCA) was used to estimate the *TDMVCL*_*MAURITIUS*_ of the 10 human life losses linked with COVID-19 in Mauritius as of 16 October 2020. The HCA model was estimated with the national life expectancy of 75.51 years and a discount rate of 3%. A sensitivity analysis was performed assuming (a) 5 and 10% discount rates, and (b) the average world life expectancy of 73.2 years, and the world highest life expectancy of 88.17 years.

**Results:** The money value of human lives lost to COVID-19, at a discounted rate of 3%, had an estimated *TDMVCL*_*MAURITIUS*_ of Int$ 3,120,689, and an average of Int$ 312,069 per human life lost. Approximately 74% of the *TDMVCL*_*MAURITIUS*_ accrued to persons aged between 20 and 59 years. Reanalysis of the model with 5 and 10% discount rates, holding national life expectancy constant, reduced the *TDMVCL*_*MAURITIUS*_ by 19.0 and 45.5%, respectively. Application of the average world life expectancy at 3% discount rate reduced *TDMVCL*_*MAURITIUS*_ by 13%; and use of the world highest life expectancy at 3% discount rate increased *TDMVCL*_*MAURITIUS*_ by 50%.

**Conclusions:** The average discounted money value per human life loss associated with COVID-19 is 12-fold the per capita GDP for Mauritius. All measures implemented to prevent widespread community transmission of COVID-19 may have saved the country 837 human lives worth Int$258,080,991. This evidence, conjointly with human rights arguments, calls for increased investments to bridge the existing gaps for achieving universal health coverage by 2030.

## Introduction

Mauritius is the second country among the 47 World Health Organization [WHO] African Region [WAFR] member states which graduated to a high-income economy. The Gross National Income (GNI) per capita for 2019 was US$ 12,740, with an estimated population of 1.27 million (1). Furthermore, Mauritius has a human development index of 0.796 and which after adjusting for inequality drops to 0.688. In the same breath, the country has a Gini index of 35.8 (2). As a result of Coronavirus Disease (COVID-19), the economy is expected to experience its first contraction in 40 years. According to the International Monetary Fund (IMF), the real gross domestic product (GDP) growth would contract by 6.8% in 2020 (3).

In 2017, which was before COVID-19 pandemic, Mauritius had a total of 10,332.65 deaths, of which 88.95% were from non-communicable diseases (NCD), 5.28% from injuries, and 5.76% from communicable diseases (CD). The death rates per 100,000 population for various CDs were: 39.12 for chronic respiratory diseases; 25.31 for respiratory infections and tuberculosis; 7.77 for maternal and neonatal disorders; 7.63 for HIV/AIDS and sexually transmitted infections; 2.19 for enteric infections; 1.08 for nutritional deficiencies; 0.14 for neglected tropical diseases and malaria; and 2.66 for other infectious diseases (4).

The COVID-19 outbreak continues to accelerate with a total of 39,175,462 confirmed cases, including 1,102,941 deaths and a case fatality rate of 2.8%, reported globally at 16 October 2020 (5). The African continent had a total of 1,621,853 cases, including 39,150 deaths and a case fatality rate (2.4%). South Africa is the hardest-hit country in the African continent and ranks eleventh globally after the United States of America (USA), India, Brazil, Russia, Spain, Argentina, Colombia, Peru, Mexico and France (5). As of 16 October 2020, there were 415 confirmed COVID-19 cases in Mauritius, including 10 deaths, 364 recovered cases, and 41 active cases (5). The case fatality rate was 2.4% and a recovery rate of 87.7% (5, 6).

Notwithstanding the growing interest for research in the area of COVID-19, there is a dearth of country evidence on the monetary value of human life losses associated with COVID-19. Brazil (7), Canada (8), China (9), France (10), Iran (11), Italy (12), Spain (13), Turkey (14), the United Kingdom [UK] (15), and the USA (16) are exceptions. Quantifying the real disease burden of COVID-19 in dollar terms is critical to building advocacy to increase investment into health-related systems. The objectives of this paper are 2-fold. First, to estimate the discounted money value of human life losses associated with COVID-19 in Mauritius as of 16 October 2020. Secondly, to estimate briefly the potential gains from preventive actions taken to contain the spread of COVID-19.

Mauritius is among the very few countries in the WAFR which has managed to halt the community transmission of COVID-19. There have been no confirmed cases of local transmission since 26 April 2020. The success might be attributed to four systemic reasons.

First, relatively good governance compared to the rest of Africa continent. The Ibrahim Index of African Governance (IIAG) is a tool for tracking African governments progress in attaining the United Nations Sustainable Development (SDG) Goal 16 relating to effective, accountable and inclusive institutions at all levels (17, 18). In 2017, Mauritius had an overall IIAG score of 79.5%, which consisted of category scores of 81.3% in safety and the rule of law (SRL), 77.2% in participation and human rights (PHR), 74.8% of sustainable economic opportunity (SEO), and 84.6% in human development (HD) (19). The Mauritius overall IIAG and the four category scores were higher than Africa's overall IIAG score of 49.9%, and category mean scores of 52.6% in SRL, 49.2% in PHR, 44.8% in SEO, and 52.8% in HD (19).

Second, Mauritius has more resourced national health system and other systems that address social determinants of health than those of many other countries in the WAFR. As shown in Table 1, the health workforce, medical devices, infrastructure, essential health service coverage, per capita current health expenditure, and safely managed water and sanitation indicators for Mauritius are significantly higher than those of the WAFR (20–25).

**Table 1:** Health system and social determinants of health indicators in Mauritius vis-à-vis the WHO African Region (WAFR).

	**Value in Mauritius**	**Average value in WAFR**
**Health workforce indicators** **(****20****)**		
Medical doctors per 10,000 population (2018)	25.3	3.0
Nursing and midwifery personnel per 10,000 population (2017)	35.2	10.1
Dentists per 10,000 population (2018)	32.8	–
Pharmacists per 10,000 population (2018)	4.2	–
**Medical devices indicators in 2013** **(****21****)**		
Computed tomography units per million population	6.4	0.4
Mammography units per million females aged 50–69 years	2.4	7.4
Radiotherapy units per million population	49.7	0.1
**Infrastructure indicators in 2013** **(****22****)**		
Specialized hospitals per 100,000 population	0.4	–
Provincial hospitals per 100,000 population	0.4	–
District/rural hospitals per 100,000 population	0.16	–
Hospitals per 100,000 population	1.0	0.8
Health centers per 100,000 population	8.84	–
Health posts per 100,000 population	0.16	–
Hospital beds per 10,000 population	34	–
**Essential health service coverage indicators in 2017** **(****23****)**		
UHC index of service coverage (SCI)	63	46
UHC SCI components: Reproductive, maternal, newborn, and child health	69	54
UHC SCI components: Infectious diseases	53	42
UHC SCI components: Non-communicable diseases	52	71
UHC SCI components: Service capacity and access	80	30
**Health financing and catastrophic out-of-pocket health spending (SDG indicator 3.8.2) in 2017**		
Population with household expenditures on health >10% of total household expenditure or income (SDG 3.8.2) (%) (24)	8.9	7.26
Population with household expenditures on health >25% of total household expenditure or income (SDG indicator 3.8.2) (%) (24)	1.8	1.78
Current Health Expenditure (CHE) per Capita in PPP (25)	1,278.01	291.9
Domestic General Government Health Expenditure as % of CHE (25)	42.87	55.52
Domestic Private Health Expenditure as % of CHE (25)	56.30	44.48
Out-of-Pocket Expenditure (OOPS) as % of CHE (25)	48.87	35.82
External health expenditure as % of CHE (25)	0.83	21.39
CHE as % Gross Domestic Product (GDP) (25)	5.72	5.65
Domestic general government health expenditure as percentage of GDP (%) (25)	2.45	1.91
**Social Determinants of Health in 2017**		
Population using safely-managed drinking-water services (%) (24, 26)	>99	29
population using safely-managed sanitation services (%) (24, 26)	96	20

The public and private health sector are manned by a total of 3,210 medical doctors (including 895 specialists), 411 dentists and 4,400 nurses and midwives (27). The doctor population ratio (25.3 per 10,000 population) and nurse and midwifery ratio (35.2 per 10,000 population) in Mauritius are, respectively, 8- and 3-fold higher than those of the WAFR (20, 22).

In 2017, Mauritius per capita current health expenditure (CHE) of US$600 (Int$1,278) was 4.4 times higher than the average of Int$292 in the AFR (25). The percentage of the population with household expenditures on health of more than 25% of the total household income increased slightly from 1.2% in 2012 to 1.8% of the population in 2018 (24, 28). In 2012, 0.34% of households were impoverished by OOP (28). The health system is adequately resourced to keep Mauritius on track to attaining the SDG 3 target 3.8 on achieving universal health coverage (UHC) (18, 29, 30). The Mauritius UHC essential health services coverage index (measured on a scale of 0 to target of 100) of 63% in 2017 was higher than the average of 46% for the WAFR (23).

Third, stronger systems that provide services related to social determinants of health. The proportion of the population using at least basic drinking-water services in Mauritius of 99% was 3-fold that of the WAFR (24, 26). Also, the proportion of the population using improved sanitation services in Mauritius of 91.0% was three-times that of WAFR in 2017 (24, 26).

Fourth, more robust disease surveillance and response system (DSRS) due to better International Health Regulation (IHR) core capacities as recommended by the 58th World Health Assembly (31, 32). In 2013, WHO developed an IHR core capacity monitoring framework consisting of a checklist and indicators that countries can use to monitor progress in the implementation of 13 IHR core capacities (33).

Table 2 shows that, except for the Zoonotic and human-animal interface, all the other 12 IHR core capacity scores for Mauritius were higher than those for the WAFR (34). The average of the 13 IHR core capacities score of 64 was higher than the average for WAFR of 44.

**Table 2:** Comparison of Mauritius and average WHO African Region IHR score per capacity, 2019.

**Core IHR capacities**	**Mauritius IHR scores**	**African Region IHR Scores**
Legislation and financing	60	43
Coordination and national focal point functions	90	51
Laboratory	53	56
Surveillance	70	61
Human resources	80	49
National health emergency framework	67	40
Health service provision	73	41
Risk communication	80	43
Points of entry	80	36
Chemical events	40	32
Radiation emergencies	40	32
Zoonotic and human-animal interface	20	50
Food safety	80	43
Average of the 13 IHR core capacities	64	44

*Source: World Health Organization [WHO] (34)*.

Despite the past relative success, the Ministry of Health and Wellness acknowledges the need to sustain advocacy for increased and efficiently utilized investments to bridge the albeit limited persisting gaps in UHC and implementation of some of the IHR core capacities (35). Card and Mooney (36) argue that given the resources available in any health system for saving life are limited, rational allocation of resources is needed, which call for monetary valuation of human life. According to Rice (37), it is important to translate adverse effects of diseases, such as COVID-19, into dollar terms which is the universal language of decision-makers in ministries of economic development, planning, and finance; the private sector; and the international development policy arena.

## Materials and Methods

### Study Area and Overview of Interventions Implemented to Combat COVID-19

The cross-sectional study reported in this paper was undertaken among the ten persons deceased due to COVID-19 in Mauritius between 18 March 2020 (when the first case was discovered) to 16 October 2020.

The Republic of Mauritius implemented a wide array of public health containment measures since the outbreak of COVID-19 was reported in the country on 18 March 2020 to prevent widespread community transmission (6, 38). These included bans on public gatherings, a curfew order, closing of borders, discontinuation of public transportation; closing of schools, universities, shopping malls, and tourist sites; suspending of employee attendance at government and private workplaces (except for essential staff); and introduction of mass testing for antigens on 27 April 2020. As the country recorded no new cases for nearly 3 weeks and no active cases since 11 May 2020, a strategically phased resumption of economic activities began on 15 May 2020. A Work Access Permit issued by the authorities, except for those working in essential sectors became mandatory for employees to resume their duties.

Arrangements were made by the public transport companies to comply with the prescribed health measures and to adhere to the physical distance between passengers. Schoolchildren had to stay at home, while the courses continued to be delivered remotely. Banks and supermarkets still operated on alphabetical order, and the same applied to post offices. Two sets of legislations were enacted mid-May 2020, namely the COVID-19 Bill and Quarantine Bill. Both legislations delineated the transition process from the curfew by strengthening the surveillance control and health system preparedness. These actions ensured a progressive reopening of economic and other activities with strict sanitary rules and added measures to avoid a resurgence of the disease (6, 38).

Notwithstanding the curfew was lifted from end of May 2020, physical distancing guidelines remain in place, as well as the mandatory wearing of masks in public. While working access permits are no longer required, offices are required to incorporate physical distancing requirements, and encourage working from home. All schools reopened in July 2020 while the borders remained closed until the end of September (6, 38).

### Empirical Framework

Every human being is imbued with unique capabilities that enable them to enjoy their right to life (Article 3), the right to rest and leisure (Article 24) (39), flourishing as a human (40), and perform expected societal roles (e.g., spouse, carer, breadwinner/worker, taxpayer, commodity consumer, investor, innovator, inventor, mentor, learner, educator, religious worshiper) (41). According to the OECD such capabilities (human capital) include “*The knowledge, skills, competencies and attributes (physical, emotional and mental health plus motivation, and behavior) embodied in individuals that facilitate the creation of personal, social and economic well-being* (p. 18)” (42). The actualization of such capabilities during one's lifetime enables the individual, the family, and the society to flourish or thrive (43). Premature death from COVID-19 (or any other cause) annihilates the stock of those embodied human capital capabilities (including health), capacity to enjoy leisure activities, ability to consume non-health goods and services, capability to contribute to government revenue (via service fees and taxes), ability to save and invest, and ability to produce goods and services for domestic use or export.

The potentially productive years of life lost [YLL] from a COVID-19 death equals the average life expectancy at birth of Mauritius minus the age of onset of death of the specific person. Jones-Lee (44) and Mooney (45) explains and discusses the strengths and limitations of the three approaches used to value monetarily statistical human life, i.e., the human capital approach (HCA), the revealed preferences approach (or implied values), and the willingness-to-pay (or contingent valuation) approach.

The current study employs the HCA originally developed by Petty (46), and after that, refined by Weisbrod (47) and Rice and Cooper (48). According to Weisbrod (47), “*The present value of a man at any given age may be defined operationally as his discounted expected future earnings stream net of his consumption*.” (p. 427). Weisbrod (47), Chisholm et al. (49), and World Health Organization [WHO] (50) recommends use of per capita GDP net of current health expenditure in the valuation of YLL.

Why use net GDP per capita, i.e., the difference between GDP per capita and health care expenditure per capita? Economic theory assumes that every rational individual strives to maximize utility (happiness or pleasure or welfare). The main direct determinants of utility are the consumption of health, non-health goods and services, and leisure (50). Individuals demand health because it is intrinsically pleasurable, allows one to engage in activities of daily living (e.g., schooling, work), and enables one to enjoy leisure activities (e.g., eating and drinking in restaurants, local and international tourism activities, sports, socializing, visiting drama and movie theaters, sports). People demand health goods and services, which do not yield utility, because of the expected positive impact on health, i.e., health-related-quality of life and length of life. Thus, the demand for health goods and services is derived from the demand for health (51). COVID-19 illness (or any other illness) compels individuals (and households) to pay for health goods and services, which reduces household disposable income, and hence, enjoyment of leisure activities and non-health goods and services that directly deliver utility (or pleasure) (49). It is for this reason that WHO (50) recommends:

“…it is important to note that GDP includes expenditure on health goods and services, so this component should be omitted, and the focus of analysis be redirected toward establishing the present value of discounted aggregate flows of current and future consumption of non-health-related goods and services linked to disease (p.4)”.

The current study replicates the HCA model developed by Weisbrod (47), and recently applied in Brazil (7), Canada (8), China (9), France (10), Iran (11), Italy (12), Spain (13), Turkey (14), the UK (15), and the USA (16) to estimate the monetary value of human lives lost due to COVID-19. The total discounted money value of human life losses linked with the 10 COVID-19 deaths in Mauritius (*TDMVCL*_*MAURITIUS*_) equals sum of the discounted money value of each case whose outcome was death (*DMVCL*_*i*_). Where ‘i' equals Case 1, Case 2, Case 3, Case 4, Case 5, Case 6, Case 7, Case 8, Case 9, and Case 10. Formulaically:

(1) $$\begin{array}{l} TDMVCL_{MAURITIUS}=\overset{CASE=10}{\underset{CASE=1}{\sum }}DMVCL_{i} \end{array}$$

The *DMVCL*_*i*_ for each i^th^ COVID-19 case with death outcome is the sum of the multiplication of discount factor, net per capita GDP for Mauritius, and years of life lost (YLL) per ith case. Where:

- Discount factor (*Q*_1_) equals $\left[\frac{1}{{\left(1+r\right)}^{t}}\right]$, *r* is the discount rate of 3% in this study (7–16, 52), and *t* is the specific YLL;
- net per capita GDP equals the difference between GDP per capita (*Q*_2_) minus current health expenditure per person (*Q*_3_) in Mauritius;
- YLL equals the average life expectancy at birth in Mauritius (*Q*_4_) minus the average age of onset of death for the i^th^ case of COVID-19 (*Q*_5_ ).

The formula for estimating *DMVCL*_*i*_ for the i^th^ case can be expressed as follows:

(2) $$\begin{array}{l} DMVCL_{i=1,..,10}=\overset{T}{\underset{t=1}{\sum }}\left(Q_{1}\right)\times \left(Q_{2}-Q_{3}\right)\times \left(Q_{4}-Q_{5}\right) \end{array}$$

Where: $\overset{t=j}{\underset{t=1}{\sum }}$ is the addition from the 1^st^ to year *T* of life for the i^*th*^ case, and the meaning of other variables are as defined earlier. 2020 was taken as the base year for the analysis.

### Data and Data Sources

The economic model (equations 1 and 2) was estimated using the following data and data sources:

- Discount rates (*Q*_1_) of 3%, 5%, and 10% from the published past COVID-19 studies (7–16).
- Data on the GDP per capita (*Q*_2_) of Mauritius of Int$26,460.581 retrieved from the IMF World Economic Outlook Database (53).
- Data on the current health expenditure per person (*Q*_3_) in Mauritius of Int$1,278.012 from the WHO Global Health Expenditure Database (25).
- Data on both sexes average life expectancy for Mauritius of 75.51 years (*Q*_4_), the world of 73.2 years, and world highest (Hong Kong Females) of 88.17 years from the Worldometer demographics data (5).
- Data on the 10 COVID-19 cases that died from the Ministry of Health and Wellness COVID-19 website (6), which is also retrievable from the Worldometer Coronavirus Pandemic Database (5).
- Data on the ages of onset of death (*Q*_5_) for persons who died of COVID-19 (i.e., Case 1 = 20 years, Case 2 = 42 years, Case 3 = 51 years, Case 4 = 59 years, Case 5 = 59 years, Case 6 = 63 years, Case 7 = 63 years, Case 8 = 69 years, Case 9 = 71 years, Case 10 = 76 years) from the Ministry of Health and Quality of Life COVID-19 database (6).
- Data on the 155 persons contaminated by 23 positive COVID-19 cases was from the Ministry of Health and Wellness (6).
- Data on Mauritian 4,500 repatriated from foreign countries, out of them 166 tested positive was from the Ministry of Health and Wellness (6).

### Data Analysis

Excel Software (Microsoft, New York) was used to analyse data following the steps below:

*Step 1:* Equations 1 and 2 in subsection Empirical Framework were built into an Excel spreadsheet.
*Step 2:* The net per capita GDP for Mauritius was estimated by subtracting current health expenditure per person from per capita GDP for Mauritius, i.e., Int$26,460.581 minus Int$1,278.012 equals Int$25,182.57.
*Step 3:* The YLL for each of the 10 COVID-19 cases that died was calculated through subtraction of the average age of onset of death from the average life expectancy at birth in Mauritius. The calculation of YLL can be illustrated using Case 1. The average age of onset of death for Case 1 was 20 years, and the average life expectancy for Mauritius was 75.51 years. Thus, the undiscounted YLL for Case 1 equals 55.51, i.e., 75.51 years minus 20 years. The undiscounted YLL for the 10 human lives lost was 187 years (See Supplementary Table 1).
*Step 4:* The discounting of YLL at 3%, 5% and 10% discount rates yielded 124, 100, and 68 years, respectively (See Supplementary Table 2).
*Step 5:* The economic model was estimated using a discount rate of 3%, which is widely applied in health-related studies (25–30, 43, 46). It entailed multiplication of the discounted YLL of 124 years by the net GDP per capita (Int$25,182.57) (See Supplementary Table 3).
*Step 6:* The average money value per COVID-19 death was calculated through the division of the total discounted money value of human lives lost in Mauritius by the total number of deaths, i.e., Int$3,120,689.13 divided by 10 deaths.
*Step 7:* The average money value per person in population was estimated through the division of the total discounted money value of human lives lost by the total population in 2020 for Mauritius, i.e., Int$3,120,689.13 divided by 1,271,766.
*Step 8:* Two univariate sensitivity analyses were conducted to test the impact of uncertainty surrounding two variables. First, due to the lack of consensus in the health economics literature, uncertainty surrounds the choice of discount rate (54, 55). In order to test the impact of changes in the discount rate on the *TDMVCL*_*MAURITIUS*_, the model was recalculated using discount rates of 5% and 10% (7–16, 56, 57). Second, there is no consensus regarding whether to apply the national average life expectancy at birth or the world highest average life expectancy at birth in the calculations of YLL (7–16). The economic model was first estimated using the national average life expectancy at birth for Mauritius, and subsequently, reanalysed with the global average life expectancy at birth and the world highest average life expectancy at birth (i.e., female average life expectancy in Hong Kong).
Step 9: The potential gains due to COVID-19 contact tracing and quarantine were estimated. The step entailed calculation of the:
  a) Case fatality rate = actual 10 COVID-19 deaths divided by total COVID-19 cases of 344 (1) = 10/344 = 0.0290697674418605.
  b) Contaminations per patient = 155 persons contaminated divided by 23 contaminators (5) = 155/23 = 6.73913043478261.
  c) Number protected by quarantine = 166 quarantined cases times contamination per patient (6.73913043478261) = 1,119.
  d) Number of COVID-19 deaths averted = 1,119 protected cases times case fatality rate (0.0290697674418605) = 33.
  e) The discounted money value of human lives saved with quarantine was equal the 33 deaths averted times the average discounted monetary value per human life of Int$312,069.
Step 10. The potential gains from all measures (which are stated in the Methods section) taken by the Government and people of Mauritius to prevent widespread community transmission of COVID-19 were estimated by multiplying the 837 predicted number of deaths from Cabore et al. (58) by average discounted money value per human life.

### Ethics Approval

Ethics approval was not necessary since the study did not involve human or animal subjects. It relied exclusively on the analysis of secondary data from IMF (53), Mauritius Ministry of Health and Wellness (6), WHO (24, 25), and Worldometer (5) databases. The data is freely accessible to the public.

## Results

### Findings of Analysis With Mauritius Life Expectancy of 75.51 Years and a 3% Discount Rate

As depicted in Table 3, the 10 human lives lost to COVID-19 had an estimated total discounted money value of Int$3,120,689, and an average of Int$312,069 per human life lost.

**Table 3:** The total discounted money value of human lives lost due to COVID-19 in Mauritius: assuming national life expectancy of 75.51 years and a discount rate of 3%.

**Case number and age at onset of death**	**Discounted money value per human life lost (Int$)**	**Percent**
Case 1: 20 years	679,060	21.8
Case 2: 42 years	532,154	17.0
Case 3: 51 years	438,508	14.1
Case 4: 59 years	331,557	10.6
Case 5: 59 years	331,557	10.6
Case 6: 63 years	267,815	8.6
Case 7: 63 years	267,815	8.6
Case 8: 69 years	156,894	5.0
Case 9: 71 years	115,329	3.7
Case 10: 76 years	0	0
Total	3,120,689	100.0
Average	312,069	

Of the total discounted money value of human lives lost due to COVID-19 (TDMVCL), 21.8% accrued to the 20 year-old case, 17.0% to the 42 year-old case, 14.1% to the 51 year-old case, 21.2% to the two 59 year-old cases, 17.2% to the two 63 year-old cases, 5.0% to the 69 year-old case, 3.7% to the 71 year-old case, and 0.0% to the 76 year-old case. The discounted money value per human life diminishes with increase in age. For instance, the discounted money value of the 20 year-old case was 6-fold higher than that of the 71 year-old case. Approximately 74.1% of the TDMVCL accrued to persons aged between 20 and 59 years, i.e., the most product age bracket.

### Findings of Reanalysis With 5 and 10% Discount Rates With Mauritius Life Expectancy of 75.51 Years

Table 4 presents the results of sensitivity analysis of using 5 and 10% discount rates.

**Table 4:** The total discounted money value of human lives lost due to COVID-19 in Mauritius: assuming 5 and 10% discount rates (in 2020 Int$).

**Case number and age at onset of death**	**Discounted money value per human life lost at 5% discount rate (Int$)**	**Discounted money value per human life lost at 10% discount rate (Int$)**
Case 1: 20 years	470,877	250,615
Case 2: 42 years	407,779	241,969
Case 3: 51 years	354,922	228,583
Case 4: 59 years	283,910	202,003
Case 5: 59 years	283,910	202,003
Case 6: 63 years	236,554	178,881
Case 7: 63 years	236,554	178,881
Case 8: 69 years	145,716	122,599
Case 9: 71 years	109,027	95,462
Case 10: 76 years	0	0
Total	2,529,250	1,700,996
Average	252,925	170,100

Reanalysis of the model with a discount rate of 5%, while holding national life expectancy constant, reduced the TDMVCL by Int$591,439 (19.0%), and the value per human life by Int$59,144. Re-estimation of the model with a 10% discount rate, holding the national life expectancy constant, decreased the TDMVCL by Int$1,419,693 (45.5%), and the value per human life by Int$141,969.

### Findings of Reanalysis With the Average Global Life Expectancy of 73.2 Years and the World Highest Life Expectancy of 88.09 Years Holding Discount Rate Constant at 3%

Table 5 portrays findings of recalculation of the economic model substituting the national life expectancy with the average world life expectancy and the world highest life expectancy.

**Table 5:** The total discounted money value of human lives lost due to COVID-19 in Mauritius—assuming average global and world's highest life expectancies (in 2020 Int$).

**Case number and age at onset of death**	**Discounted money value per human life lost at average global life expectancy of 73.2 years and 3% discount rate (Int$)**	**Discounted money value per human life lost at average world highest life expectancy of 88.09 years and 3% discount rate (Int$)**
Case 1: 20 years	664,190.0	726,946
Case 2: 42 years	503,662.2	623,909
Case 3: 51 years	401,332.5	558,228
Case 4: 59 years	284,464.2	483,215
Case 5: 59 years	284,464.2	483,215
Case 6: 63 years	214,812.4	438,508
Case 7: 63 years	214,812.4	438,508
Case 8: 69 years	93,606.1	360,710
Case 9: 71 years	48,186.1	331,557
Case 10: 76 years	0	250,667
Total	2,709,530	4,695,463
Average	270,953	469,546

Application of the average world life expectancy of 73.2 years, with a 3% discount rate, slashed the TDMVCL by Int$411,159 (13%), and the value per human life by Int$41,116. Recalculation of the model with the highest life expectancy in the world of 88.09 years, holding discount rate constant at 3%, enlarged the TDMVCL by Int$1,574,773 (50%), and the average discounted money value per human life by Int$157,477.

### Potential Gains From COVID-19 Contact Tracing and Quarantine

Without contact tracing and quarantine, a total of 43 persons would have died due to COVID-19 with a monetary value of Int$13,269,240, i.e., Int$10,148,550 (value of 33 averted deaths) plus Int$3,120,689 (value of 10 actual dead cases). Therefore, quarantine helped Mauritius to save 33 human lives with a discounted monetary value of Int$10,148,550, i.e., the 33 deaths averted times average discounted monetary value of Int$312,069 per human life.

### Potential Gains From All Measures Taken by the Republic of Mauritius to Prevent Widespread Community Transmission of COVID-19

The widespread community transmission of COVID-19 infection in Mauritius, as predicted by Cabore et al. (58) would have led to a total of 837 losses in human lives with a total discounted monetary value of Int$261,201,681, i.e., 837 deaths times average discounted money value of Int$312,069 per human life. Thus, all measures implemented in the Republic of Mauritius to prevent widespread community transmission of COVID-19 may have saved the country a total of Int$258,080,991, i.e., Int$261,201,681 minus Int$3,120,689 (total value of the actual 10 deaths).

## Discussion

### Key Findings and Implications

- The 10 human lives lost to COVID-19 had an estimated total discounted money value of Int$3,120,689.
- The average discounted money value per human life was Int$312,069.
- Reanalysis of the model with discount rates of 5 and 10% attenuated the TDMVCL by 19.0 and 45.5%, respectively.
- The application of the average global life expectancy of 73.2 years slashed the TDMVCL by 13%.
- The use of highest life expectancy in the world of 88.09 years enlarged the TDMVCL by 50%.
- Quarantine saved 33 human lives with a discounted monetary value of Int$10,148,550.

All measures implemented to prevent widespread community transmission of COVID-19 may have saved the country 837 human lives worth Int$258,080,991. The economic impacts of COVID-19, as well as the implementation of related containment public health measures, are well-determined. In April 2020 the IMF forecasted that the national economy would contract by 6.8% in 2020. As long as other countries are not COVID-19 free, Mauritius, which is an economy heavily dependent on the tourism industry remains vulnerable to the Specter of the global pandemic. In the same vein, the national debate whether to open the borders to give some breathing space and to allow the tourism industry to remain afloat financially is high on the agenda. However, as the threat of the pandemic still looms, the economic loss due to public health measures should be weighed against the potential gains estimated at Int$261,201,681. While containment measures come at a cost and stop in economic activity, beyond economics, the priority should be on the impact on length and quality of life of people.

The TDMVCL was 0.009% of the total GDP (in PPP) for Mauritius in 2020. Whereas, the average discounted money value per human life loss associated with COVID-19 was 12-fold the per capita GDP for Mauritius.

An increase in the discount rate from 3 to 10% results in a drop in TDMVCL from Int$3,120,689 to Int$1,700,996. This represents a 46% decrease. Also, a 16.7% growth in the average life expectancy at birth leads to an expansion in TDMVCL of 50%. This result confirms the findings from past studies that indeed, the magnitude money value of human life losses is dependent on both the discount rate and the average life expectancies used (7–16).

### Comparison With Other Studies

Table 6 provides a comparison of the findings from the Mauritius study with those of 10 other countries that employed the HCA to estimate the monetary value of human life losses associated with COVID-19.

**Table 6:** A comparison of Mauritius discounted money value of human life losses associated with COVID-19 to those of 10 other countries.

**Countries**	**Total discounted money value of human life losses**	**The average discounted money value per human life**
Mauritius*	Int$3,120,689	Int$312,069
Brazil (7)	Int$3,591,028,164	Int$99,629
Canada (8)	Int$2,037,021,173	Int$231,217
China (9)	Int$924,346,795	Int$356,203
France (10)	Int$10,492,290,194	Int$339,381
Iran (11)	Int$3,510,063,043	Int$165,187
Italy (12)	Int$13,070,141,190	nt$369,088
Spain (13)	Int$9,629,234,112	Int$470,798
Turkey (14)	Int$1,098,469,122	Int$228,514
United Kingdom [UK] (15)	Int$9,883,426,226	Int$225,104
United States of America [USA] (16)	Int$19,780,290,991	Int$292,889

Sources: *Estimates from the current study. Sources for other countries are referenced in the Table.

The total discounted money value of human life losses in Mauritius was 1,151-fold lower than those of Brazil (7); 653-fold of Canada (8); 296-fold of China (9); 3,362-fold of France (10); 1,125-fold of Iran (11); 4,188-fold of Italy (12); 3,086-fold of Spain (13); 352-fold of Turkey (14); 3,167-fold of the UK (15); and 6,338-fold of the USA (16). The differences could be attributed to significantly lower number of COVID-19 deaths in Mauritius compared to the six other countries.

The average discounted money value per human life in Mauritius was higher than the other six countries (Brazil, Canada, Iran, Turkey, UK, and USA), which is related to higher mortality rate in the younger age groups in Mauritius. As the share of deaths in Canada, France, Italy, Turkey, UK and USA in the older age group of 60 years and above was considerably much higher, the component of years' life lost (life expectancy less age at onset of death due to COVID-19) is lower. Conversely, as people died from COVID-19 at relatively younger age in China and Spain, the benefits/returns foregone had these people stayed alive are much higher per person in China and Spain compared to Mauritius.

### Strengths of the Study

This study applied HCA, a well-known economic methodology, to monetarily value the human life losses associated with COVID-19 in Mauritius. It is the first study of its kind in Mauritius. The evidence presented in this paper can be judiciously used by the Ministry of Health and Wellness to make a case for augmenting investments to strengthen health-related systems to bridge extant service coverage gaps. Universal coverage of health and health-related services would contribute in assuring every citizen's right to life, and achievement of the SDG3 on “Ensuring healthy lives and promote well-being for all at all ages” and SDG6 on “Ensuring availability and sustainable management of water and sanitation for all” [(18), p. 14].

### Limitations of the Study

The study had some shortcomings. First, the scope of the study was limited to the impact of COVID-19 on the life expectancy of the ten persons who died. Therefore, since the study did not evaluate both the costs and consequences of alternative COVID-19 control intervention options, the study findings can only be used for advocacy and not to inform policy development and decision-making.

Second, the study did not include the cost of societal resources expended in prevention (water, sanitation, handwashing with soap, hand-sanitisers, facial masks, personal protective equipment for health workers), quarantine, testing, contact-tracing, treatment, and rehabilitation of the 332 cases that recovered from COVID-19 infection. It did not also include the cost of diagnosis, treatment, post-mortem, mortuary storage, and interment of the 10 cases that died.

Third, Santarpia et al. (59) conducted a study among 13 individuals with COVID-19 isolated at the University of Nebraska Medical Center to examine aerosol and surface contamination with SARS-CoV-2. The authors found that “…*data indicate significant environmental contamination in rooms where patients infected with SARS-CoV-2 are housed and cared for, regardless of the degree of symptoms or acuity of illness. Contamination exists in all types of samples: high and low-volume air samples, as well as surface samples including personal items, room surfaces, and toilets*” (p.3). This implies that since there may still be aerosol and surface contamination at quarantine and isolation centers, we may have overestimated the effect of quarantine, and hence, the potential discounted money value of human lives saved.

Fourth, the HCA approach used has a number of weaknesses: (a) it uses GDP per capita to value the YLL, which ignores non-market contributions to societal welfare, the negative impact of economic production processes (e.g., on climate change), inequalities in the distribution of wealth and income, and quality of life (60); (b) values the YLL above the national average life expectancy at birth at zero; (c) assumes that the only objective of improving (or sustaining) human health (health-related quality and length of life) is to contribute to economic production (61), which disregards other objectives such as assuring human rights (39), and enabling homo sapiens to flourish (40).

## Conclusion

This study succeeded in estimating the discounted money value of human life losses associated with COVID-19 in Mauritius as of 16 October 2020. The average discounted money value per human life loss associated with COVID-19 of Int$312,069 is significant, since it is 12-fold the per capita GDP for Mauritius. As noted earlier, the Republic of Mauritius prompt action in arresting the spread of COVID-19 infections, optimizing recoveries, and limiting the number of deaths is laudable. All measures implemented to prevent widespread community transmission of COVID-19 may have saved the country 837 human lives worth Int$258,080,991. This effectiveness has been attributed to relatively good political governance, and a highly performing national health system, disease surveillance and response system, and other systems that address social determinants of health.

The Ministry of Health and Wellness can use the evidence contained in this paper, conjointly with human rights (to life, health, and health care) arguments, to sustain advocacy for further increase in multisector investments to bridge the existing limited gaps in UHC, IHR core capacities, and social determinants of health to mitigate and to respond to future public health emergencies, and to sustain the good health indicators.

In order to guide decision-making related to COVID-19, there is a need for studies that estimate both costs and consequences of alternative prevention (e.g., lockdown, handwashing, physical distancing), contact-tracing, quarantine, treatment, and rehabilitation interventions (62, 63).

## Data Availability Statement

The original contributions presented in the study are included in the article/Supplementary Materials, further inquiries can be directed to the corresponding author/s.

## Author Contributions

LM, AN, and JK designed the study, extracted the data on per capita GDP from IMF database, current health expenditure per person from WHO Global Health Expenditure database, number of COVID-19 deaths in Mauritius from the Worldometer database, ages of onset of death from the Republic of Mauritius Ministry of Health and Wellness website, developed the human capital approach model on Excel software, and wrote the manuscript. All authors have read and agreed to the published version of the manuscript.

## Conflict of Interest

LM and AN are current employees of the WHO. However, the employer did not influence the conduct and outcome of the study in anyway. The remaining author declares that the research was conducted in the absence of any commercial or financial relationships that could be construed as a potential conflict of interest.
