# Supplementary material for: The Discounted Money Value of Human Life Losses Associated With COVID-19 in Mauritius
Source: Front Public Health. 2020 Nov 10;8:604394. doi: 10.3389/fpubh.2020.604394 (PMC7683431; doi:10.3389/fpubh.2020.604394)
Supplement: Supplementary file 1 [file Table_1.DOCX]

Supplementary Material

# Supplementary Tables

## Supplementary Tables

**
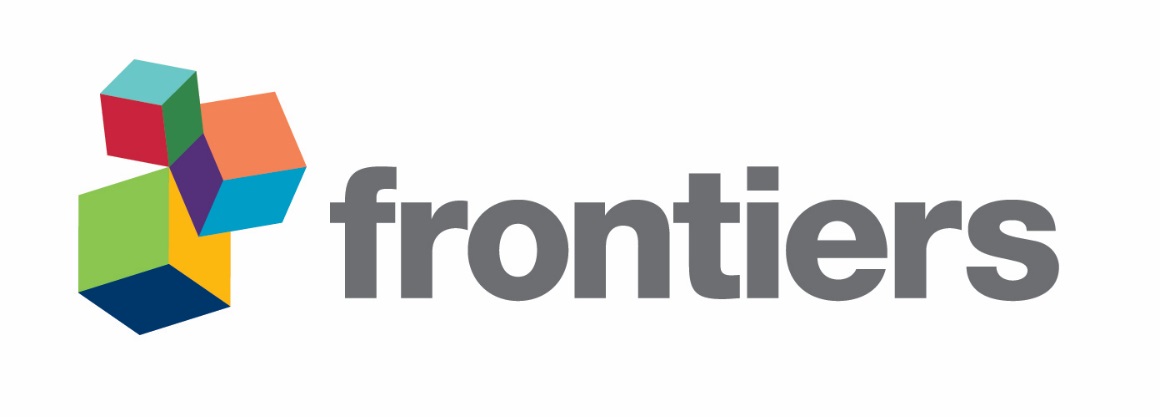
**

**Supplementary Table 1. Undiscounted potential years of life (YLL) lost due to COVID-19 in Mauritius**

| Cases | Average life expectancy at birth (in years) for Mauritius (A)^*^ | Age at onset of death (B)^**^ | Undiscounted years of life lost (C)  [C=A-B]^***^ |
| --- | --- | --- | --- |
| Case 1 | 76 | 20 | 56 |
| Case 2 | 76 | 42 | 34 |
| Case 3 | 76 | 51 | 25 |
| Case 4 | 76 | 59 | 17 |
| Case 5 | 76 | 59 | 17 |
| Case 6 | 76 | 63 | 13 |
| Case 7 | 76 | 63 | 13 |
| Case 8 | 76 | 69 | 7 |
| Case 9 | 76 | 71 | 5 |
| Case 10 | 76 | 76 | 0 |
| TOTAL |  |  | 187 |

Source: ^*^Worldometer (1). ^**^Ministry of Health and Wellness (5). ^***^Authors estimates.

**Supplementary Table 2. Discounted potential years of life lost due to COVID-19 in Mauritius**

| Cases | Undiscounted years of life lost (YLL) | YLL discounted at 3% | YLL discounted at 5% | YLL discounted at 10% |
| --- | --- | --- | --- | --- |
| Case 1 | 56 | 26.96546373 | 18.69854473 | 9.951914999 |
| Case 2 | 34 | 21.13183668 | 16.19290401 | 9.60857487 |
| Case 3 | 25 | 17.41314769 | 14.09394457 | 9.077040018 |
| Case 4 | 17 | 13.16611847 | 11.27406625 | 8.021553311 |
| Case 5 | 17 | 13.16611847 | 11.27406625 | 8.021553311 |
| Case 6 | 13 | 10.63495533 | 9.393572987 | 7.103356203 |
| Case 7 | 13 | 10.63495533 | 9.393572987 | 7.103356203 |
| Case 8 | 7 | 6.230282955 | 5.786373397 | 4.868418818 |
| Case 9 | 5 | 4.579707187 | 4.329476671 | 3.790786769 |
| Case 10 | 0 | 0 | 0 | 0 |
| **TOTAL** | **187** | **124** | **100** | **68** |

Source: Authors estimates.

**Supplementary Table 3. Calculations of the discounted money value of human life losses associated with COVID-19 in Mauritius as of 25 July 2020**

| Cases | (A) Undiscounted years of life lost (YLL) | (B) Net per capita GDP (Int$) | (C) Undiscounted money value per human life (Int$) [C=A x B] | (D) YLL discounted at 3% | (E) Money value per human life discounted at 3% [E=B x D] | (F) YLL discounted at 5% | (G) Money value per human life discounted at 5% [G=B x F] | (H) YLL discounted at 10% | (I) Money value per human life discounted at 10% [I=B x H] |
| --- | --- | --- | --- | --- | --- | --- | --- | --- | --- |
| Case 1 | 56 | 25,182.57 | 1,410,224 | 26.96546373 | 679,060 | 18.698544730 | 470,877 | 9.951914999 | 250,615 |
| Case 2 | 34 | 25,182.57 | 856,207 | 21.13183668 | 532,154 | 16.192904010 | 407,779 | 9.608574870 | 241,969 |
| Case 3 | 25 | 25,182.57 | 629,564 | 17.41314769 | 438,508 | 14.093944570 | 354,922 | 9.077040018 | 228,583 |
| Case 4 | 17 | 25,182.57 | 428,104 | 13.16611847 | 331,557 | 11.274066250 | 283,910 | 8.021553311 | 202,003 |
| Case 5 | 17 | 25,182.57 | 428,104 | 13.16611847 | 331,557 | 11.274066250 | 283,910 | 8.021553311 | 202,003 |
| Case 6 | 13 | 25,182.57 | 327,373 | 10.63495533 | 267,816 | 9.393572987 | 236,554 | 7.103356203 | 178,881 |
| Case 7 | 13 | 25,182.57 | 327,373 | 10.63495533 | 267,816 | 9.393572987 | 236,554 | 7.103356203 | 178,881 |
| Case 8 | 7 | 25,182.57 | 176,278 | 6.230282955 | 156,895 | 5.786373397 | 145,716 | 4.868418818 | 122,599 |
| Case 9 | 5 | 25,182.57 | 125,913 | 4.579707187 | 115,329 | 4.329476671 | 109,027 | 3.790786769 | 95,462 |
| Case 10 | 0 | 25,182.57 | - | 0 | 0 | - | - |  | - |
| TOTAL | 187 |  | 4,709,141 | 124 | 3,120,689 | 100 | 2,529,250 | 68 | 1,700,996 |
| Average money value per human life (Int$) |  |  | 470,914 |  | 312,069 |  | 252,925 |  | 170,100 |
| Average money value per person in population (Int$) |  |  | 3.70 |  | 2.45 |  | 1.99 |  | 1.34 |

Source: Authors estimates.
